# Equivalent titanium dioxide nanoparticle deposition by intratracheal instillation and whole body inhalation: the effect of dose rate on acute respiratory tract inflammation

**DOI:** 10.1186/1743-8977-11-5

**Published:** 2014-01-24

**Authors:** Brittany L Baisch, Nancy M Corson, Pamela Wade-Mercer, Robert Gelein, Andrea J Kennell, Günter Oberdörster, Alison Elder

**Affiliations:** 1 Department of Environmental Medicine, University of Rochester, School of Medicine and Dentistry, 601 Elmwood Avenue, Box 850, Rochester, NY 14642, USA

**Keywords:** Dose rate, Nanoparticle, Titanium dioxide, Respiratory toxicology, Whole body inhalation, Intratracheal instillation, Acute inflammation, Bronchoalveolar lavage

## Abstract

**Background:**

The increased production of nanomaterials has caused a corresponding increase in concern about human exposures in consumer and occupational settings. Studies in rodents have evaluated dose–response relationships following respiratory tract (RT) delivery of nanoparticles (NPs) in order to identify potential hazards. However, these studies often use bolus methods that deliver NPs at high dose rates that do not reflect real world exposures and do not measure the actual deposited dose of NPs. We hypothesize that the delivered dose rate is a key determinant of the inflammatory response in the RT when the deposited dose is constant.

**Methods:**

F-344 rats were exposed to the same deposited doses of titanium dioxide (TiO_2_) NPs by single or repeated high dose rate intratracheal instillation or low dose rate whole body aerosol inhalation. Controls were exposed to saline or filtered air. Bronchoalveolar lavage fluid (BALF) neutrophils, biochemical parameters and inflammatory mediator release were quantified 4, 8, and 24 hr and 7 days after exposure.

**Results:**

Although the initial lung burdens of TiO_2_ were the same between the two methods, instillation resulted in greater short term retention than inhalation. There was a statistically significant increase in BALF neutrophils at 4, 8 and 24 hr after the single high dose TiO_2_ instillation compared to saline controls and to TiO_2_ inhalation, whereas TiO_2_ inhalation resulted in a modest, yet significant, increase in BALF neutrophils 24 hr after exposure. The acute inflammatory response following instillation was driven primarily by monocyte chemoattractant protein-1 and macrophage inflammatory protein-2, mainly within the lung. Increases in heme oxygenase-1 in the lung were also higher following instillation than inhalation. TiO_2_ inhalation resulted in few time dependent changes in the inflammatory mediator release. The single low dose and repeated exposure scenarios had similar BALF cellular and mediator response trends, although the responses for single exposures were more robust.

**Conclusions:**

High dose rate NP delivery elicits significantly greater inflammation compared to low dose rate delivery. Although high dose rate methods can be used for quantitative ranking of NP hazards, these data caution against their use for quantitative risk assessment.

## Background

Nanoparticles (NPs) have diverse applications that have proven to be beneficial to society. They are used in many industrial processes (e.g., electronic, optical, mechanical) for the manufacture of composite materials such as semiconductors and solar cells. They are also ingredients in sunscreens and cosmetics, additives in odor-resistant clothing, and filters for soil and water remediation. In the past decade, nanomaterials have emerged as pharmaceutical carriers and imaging agents in the biomedical field [1,2]. In fact, there are over 1,300 nanomaterial-containing products on the market today [3]. The increased production, consumer use and disposal of NP-containing products have led to a corresponding increase in the potential for accidental or incidental human exposures via the inhalation, dermal and ingestion routes as well as concerns about environmental impacts. Indeed, nanotoxicological research is focused on identifying and characterizing the hazards associated with NPs with an overall goal of generating meaningful data for regulatory purposes.

Metal oxide NPs are of specific interest since some, such as titanium dioxide (TiO_2_), are amongst the most widely used NPs, produced in large volumes, and have been commercially available in several shapes and sizes for decades. The present study utilizes Evonik TiO_2_, which has been used extensively in the toxicological literature and has been previously well characterized [4,5]. For example, *in vivo* studies have shown that subchronic and chronic inhalation exposures to high concentrations of nanosized TiO_2_ leads to lung inflammation, increased epithelial cell proliferation, and even lung tumors in rats [6-9]. These studies also provided evidence that nanosized TiO_2_ was more potent than larger TiO_2_ particles when the deposited mass doses were similar.

Many current experimental approaches for assessing hazards from exposure to airborne nanoparticles use high dose rate delivery (bolus exposure) combined with high doses of particles. This does not reflect real-world exposure conditions and can lead to overestimation of hazard [10,11]. Intratracheal instillation is one such bolus delivery technique, whereby NPs are suspended in liquid and rapidly delivered to the tracheobronchial and alveolar regions of the respiratory tract (RT) of anesthetized animals, and which results in uneven distribution of the material [10,12]. Inhalation exposure, on the other hand, is the gold standard for RT delivery of airborne NPs for toxicity assessments, but is technically challenging and requires large amounts of material. The greatest difference between these bolus and aerosol delivery methods is the deposited dose rate of the NPs to the RT.

We hypothesize that the deposited dose rate is a key determinant of the acute inflammatory response in the RT. In the present study, dose rate was varied while keeping the deposited dose constant. Different exposure methods were used to vary dose rate: intratracheal instillation was the high dose rate delivery, which occurred within a fraction of a second (~0.5 sec), and whole body inhalation was the low dose rate delivery, which occurred over 4 hr. We also varied the dose rate by employing repeated exposure scenarios, which fractioned the same deposited dose over 4 days. Cellular and biochemical markers of acute lung inflammation and the levels of mediators that influence the progression and resolution of the inflammatory response were assessed. We demonstrate a higher inflammatory response following intratracheal instillation compared to whole body inhalation for single and repeated exposures when deposited doses were held constant. Although we did not evaluate the predictive power of intratracheal instillation for NP risk assessment, our study reinforces the need to carefully consider the use of bolus, high dose rate delivery methods for risk characterization.

## Results and discussion

The unique aspect of our study design was that the same deposited doses were achieved via intratracheal instillation and whole body inhalation, which allowed us to directly compare the inflammatory responses on the basis of deposited dose rate. This is important not only because the dose itself can determine the mechanism of response for soluble compounds [13], but we also suggest the dose rate and, therefore, underlying mechanisms may be different between the two exposure methods for NPs.

### Characterization of TiO_2_ size distributions in air and saline

#### Aerodynamic properties of TiO_2_ NPs

The size distributions of the material were assessed in air and saline for inhalation and instillation exposures, respectively (Table 1). The aerosol mass median aerodynamic diameter (MMAD) of 800 nm (geometric standard deviation, GSD 1.4) and count median diameter (CMD) of 900 nm (1.8 GSD) were very similar and together indicate that TiO_2_ NPs agglomerated in air. This is consistent with reports that aerosolized TiO_2_ tends to agglomerate to ~0.7-1.5 μm in air whether the material has a primary size in the micrometer range [7,14-16] or in the nanometer range [6,8,9]. The aerosol diameters were the same at concentrations of 33 ± 4 mg/m^3^ or 13 ± 1 mg/m^3^ and over the course of the exposures, indicating that the animals were exposed to similarly sized agglomerates.

**Table 1:** **The size distribution characteristics of TiO**_**2 **_**in air and saline**

**Medium**	**Concentration**	**Size distribution**
Air	33 ± 4 mg/m^3^	MMAD 800 nm, 1.4 GSD
		CMD 900 nm, 1.8 GSD
Saline	80 μg/mL	DLS z-avg 1350 nm, peak range 800–1700 nm
		LDS mean 6363 nm, peak range 900–11000 nm

MMAD, mass median aerodynamic diameter; CMD, count median diameter; GSD, geometric standard deviation; DLS, dynamic light scattering (intensity mode); LDS, laser diffraction spectroscopy (volume-based). DLS and LDS are representative of 3 separate runs.

#### ***Hydrodynamic properties of TiO_2_ NPs***

For instillation exposures, we assessed the hydrodynamic diameter of TiO_2_ in saline within 15 min after it was indirectly sonicated (cup horn) for 5 sec; all suspensions were additionally vortexed for 30 sec immediately prior to measurement. The suspension had an intensity-based average hydrodynamic diameter (z-average) of 1350 nm, with a single, broad peak with values ranging from 800–1700 nm when assessed by dynamic light scattering (DLS) (Table 1). Although DLS is a commonly used technique, it has disadvantages such as bias toward smaller particles and it requires that suspensions be substantially diluted [17]. Indeed, the TiO_2_ NP suspensions had to be diluted 1:10 in order to obtain size distribution data, as the measurement of the undiluted instillate was not possible. For these reasons, we also assessed the hydrodynamic diameter using laser diffraction spectroscopy (LDS) to determined the volume-based size distribution of diluted suspensions (1:10) and the mean was greater than 6 μm with values ranging from 0.9–11 μm (Table 1), although 10% of the sample was a separate, broad peak at ~9 μm (*data not shown*). Importantly, by either DLS or LDS, similar hydrodynamic diameters were observed for up to 1 hr post sonication (*data not shown*), indicating that the agglomeration state of the material was consistent for each animal that was instilled. We also assessed the hydrodynamic diameter for the low dose studies and the repeated exposure studies and found them to be consistent with size distributions as reported in Table 1 (*data not shown*). The DLS and LDS measurements cannot be directly compared because LDS measures across a wider range of particle sizes (10 nm to thousands of nm) and does not neglect particle settling or the presence of large agglomerates [18]. Likewise, it is not possible to directly compare hydrodynamic and aerodynamic particle size distributions because of inherent differences in analytical tools and in the concentrations of airborne and saline suspended NPs.

These data regarding airborne particles or liquid suspension of particles do not address the size distribution of the particles in the lung upon deposition and interactions with lung lining fluid. We presume that the size distribution of the material within the lung may differ based on delivery method from previous work [10,12,19]. These studies showed that intratracheal instillation results in more proximal and uneven distribution in the lung, whereas whole body inhalation results in more even distribution within the lung, including deposition to more distal regions and some carinal hotspots [20]. In fact, the inflammatory cell influx following deposition of similar lung burdens of fine and ultrafine TiO_2_ were higher following intratracheal instillation compared to intratracheal inhalation exposure in a study by Osier and Oberdörster [21], although inflammatory mediator release was not assessed. These differences were attributed to both differences in dose rate and unevenness of distribution. Furthermore, Henderson et al. [22] reported greater inflammatory effects of similar lung burdens of α-quartz 1 week following repeated inhalation compared to single instillation, where the inhalation animals were subjected to whole lung lavage and the instilled animals only had right lobes lavaged. Therefore, potential response differences due to uneven distribution of the material were not taken into account. Since unevenness of distribution may have played a role in response outcomes in our study, we employed whole-lung lavage. However, it is still unclear if unevenness of deposition alone is linked to mechanistic differences in response; further investigation would be beyond the scope of this manuscript.

### Dosimetry

#### ***Initial lung burden of TiO_2_ NPs***

The first aim of these studies was to achieve the same initial lung burden (ILB) into the lower RT for both intratracheal instillation and whole body inhalation exposures. Several studies have compared inhalation to intratracheal instillation [22-26] or with other bolus delivery methods, such as pharyngeal aspiration [27,28]. However, these studies reported estimated deposited doses without confirming them. Because model estimates can deviate significantly from actual values, we measured and used for comparison the actual deposited doses in unlavaged lungs immediately following exposure (Table 2). Whole body inhalation ILBs were not found to be statistically significantly different from intratracheal instillation ILBs; high dose single and repeated exposures also had similar deposited doses of TiO_2_. We assume that the TiO_2_ we quantified in the lung was in the particulate form based on results from a 7 day dissolution experiment, where levels of soluble Ti were below the instrument level of detection (*data not shown*). The poor solubility of TiO_2_ has been well recognized for decades, although recent findings by Al-Jubory and Handy [29] suggested that nanosized TiO_2_ NPs release up to 6.9 μg Ti/hr in simulated piscine intestinal fluid.

**Table 2:** **Initial lung burdens of TiO**_**2**_

**Exposure type**	**Whole body inhalation**	**Intratracheal instillation**
**Single low dose (μg)**	44.56 ± 2.85	39.24 ± 1.04
**Single high dose (μg)**	170.25 ± 9.15	163.58 ± 3.84
**Repeated high dose (μg)**	197.89 ± 8.89	179.61 ± 17.14

Values are group means (n = 3–5) of NP mass doses (μg) ± standard deviation (SD) immediately following instillation and 4 hr inhalation exposures.

Our dosimetry findings reinforce the need to be explicit about expressing dose to the lung. For example, Mizuguchi et al. [30] described the “deposited dose” of NiO in their instillation/inhalation comparison study and Yamamoto et al. [31] quantified the dose of potassium hexatitanate that was instilled or inhaled; however, in both of these studies, measurements were performed 3 days after exposure. Such measurements characterize short term alveolar retention of the material, not ILB.

#### ***Short term retention of TiO_2_NPs***

**Figure 1:**
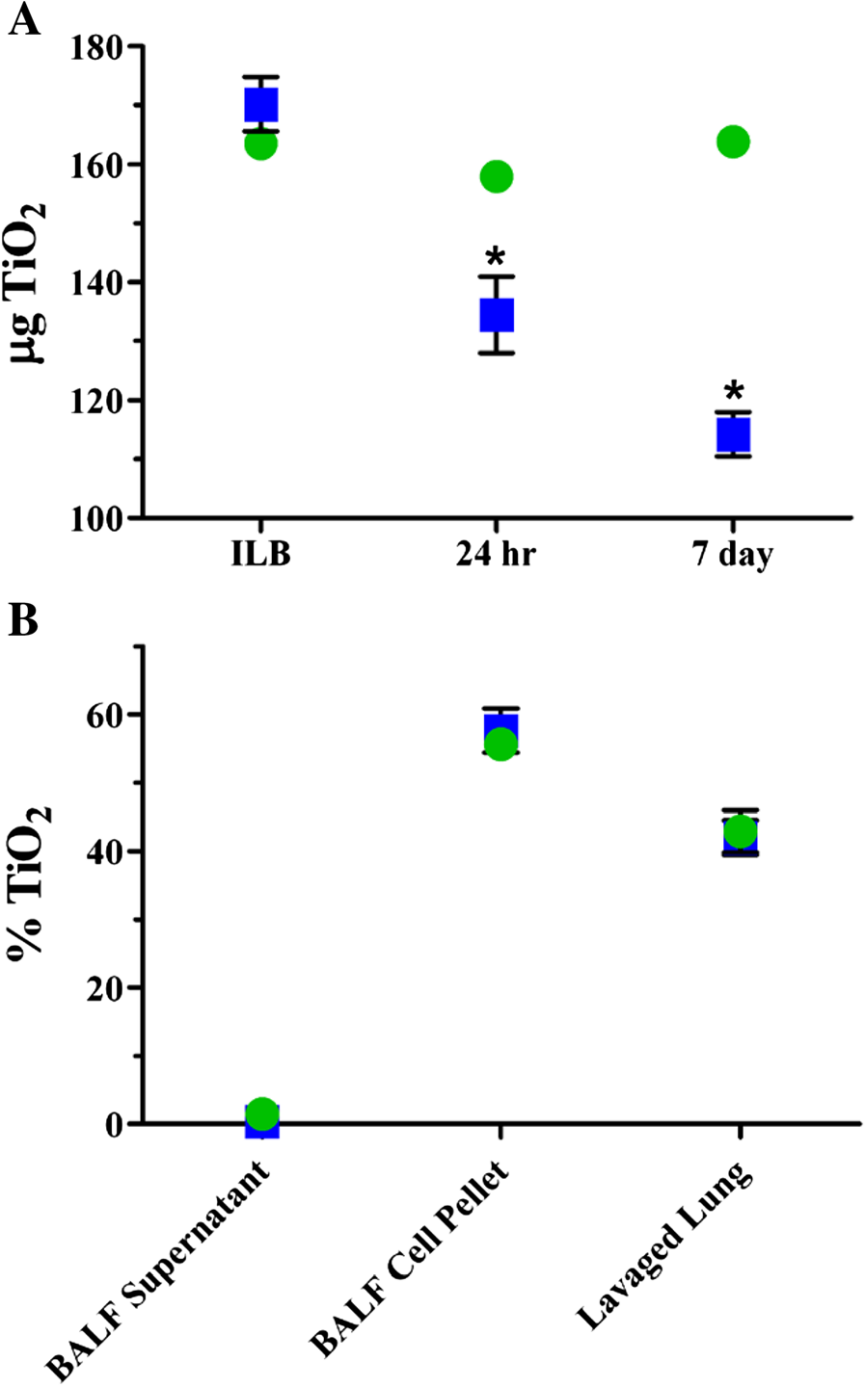
**Dosimetry and short term retention of TiO**_**2 **_**for single high dose TiO**_**2 **_**exposure.** The mass of TiO_2_ deposited in the lung following whole body inhalation (*blue squares*) and intratracheal instillation (*green circles*) was determined immediately, 24 hr and 7 days post exposure **(A)** and the compartmentalization of TiO_2_ in BALF supernatant, BALF cell pellet and the remaining lung tissue after lavage (lavaged lung) were determined 24 hr post exposure **(B)**. Values are group means (n = 3–5) ± standard error (SE). The inhalation and instillation ILBs were not found to be significantly different; *, significantly different compared to the respective ILB (p < 0.05).

Short term retention was evaluated over a 7 day period following the single, high dose exposures in lungs that were not lavaged (Figure 1A). Although the instillation and inhalation ILBs were similar, the short term retention patterns were different, with statistically significant decreases over time following inhalation, but little change for instilled animals. Within the first 24 hr following inhalation exposure, clearance is likely due to the mucociliary transport of the deposited TiO_2_ NPs from the tracheobronchial region and the posterior portions of the nasal passages [32]. At 7 days, clearance is likely from the alveolar region. Some studies have suggested that deagglomerated, nanosized TiO_2_ can reach the interstitium and, potentially, the vasculature during the clearance process [7,33-38]. We did not detect TiO_2_ in the blood at 24 hr or 7 days post exposure (*data not shown*) and translocation to secondary organs was not evaluated in this study.

**Figure 2:**
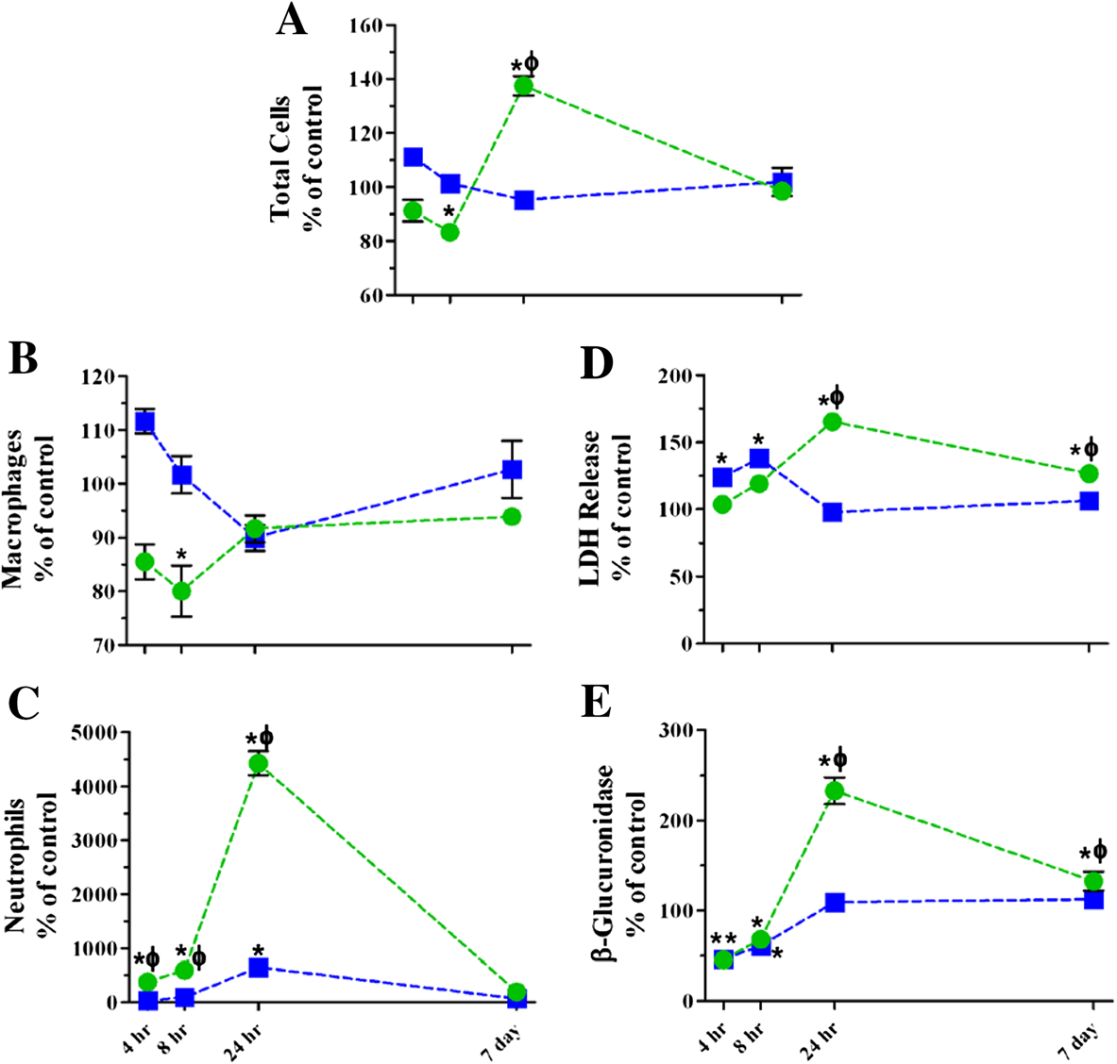
**Time course of changes in BALF cellular and biochemical parameters following single high dose exposure to TiO**_**2 **_**NPs.** The numbers of cells **(A)**, macrophages **(B)**, and neutrophils **(C)**, and LDH **(D)** and β-glucuronidase activities **(E)** were assessed 4, 8, 24 hr and 7 days post inhalation (*blue squares*) or instillation (*green circles*) exposure. Values are group means ± SE (n = 5) and are represented as a percentage of respective controls. *, significantly different from corresponding controls; Φ, significantly different between exposure methods (p < 0.05).

Even though the clearance of particles from the alveolar region would begin immediately after exposure, it would take several months for the particles to fully clear from the RT [32] (T_1/2_ ~70 days). We assume that the short term retention profiles for the two exposure methods are different because the dissimilar deposition patterns, higher local dose per unit time, and the ensuing inflammation following instillation of TiO_2_ (Figure 2C) may have perturbed the clearance. Furthermore, previous findings demonstrated that over several months, the long term clearance patterns (> 7 days) are similar for the two exposure methods, both at low and high lung TiO_2_ burdens [39].

#### ***Compartmentalization of TiO_2_ NPs in BALF supernatant, BALF cell pellet and lavaged lung tissue***

We assessed the compartmentalization of deposited TiO_2_ in the RT, namely in BALF supernatant, pellet and lavaged lung 24 hr post exposure (Figure 1B). The compartmentalization patterns of the instilled and inhaled animals are identical, with the majority of the material being associated with lavaged cells or lung parenchyma (inside or firmly attached to cells). Ferin et al. [40] also reported similar findings, where ~69% of instilled ultrafine TiO_2_ was recovered in the BALF pellet and ~30% in the remaining lung tissue. Geiser et al. [37] documented similar findings at 1 hr and 24 hr post inhalation of ultrafine TiO_2_ in rats: on average, ~79% of the particles were on the luminal side of the airway and alveolar surfaces, ~5% were inside epithelial or endothelial cells, ~5% in connective tissues and ~11% in capillary lumens. Kapp et al. [36] also reported TiO_2_ internalization in type I pneumocytes.

Within the BALF pellet, we expect that the material is associated with macrophages, specifically, given that with 5 lavages the majority of the extracted cells (~60%) are macrophages (Figure 2B) [34]. This would therefore leave ~40% of the macrophages in the lung, meaning that they are still a predominant cell type in the alveolar sacs, likely interacting with the TiO_2_. Other studies have indicated that physical interactions between nanomaterials and cells are necessary in order to elicit or enhance an inflammatory response or mediator release [41-43], and numerous *in vitro* studies have demonstrated that uptake and mechanisms may be cell type dependent. Our data show that the TiO_2_ NPs were primarily cell associated (inside or firmly attached to cells) and elicited a significant, acute inflammatory response in the RT *in vivo*.

### Lung inflammatory responses following single exposure to high and low doses of TiO_2_ NPs

#### ***Impact of dispersant on TiO_2_ NP-induced neutrophil influx***

Many *in vitro* and *in vivo* studies use coatings, such as surfactants, in order to mimic the lung lining fluid in an *in vivo* scenario [44] and/or to obtain monodisperse, stabilized suspensions [45]. Here, we did not use coatings because: 1) real world RT exposures do not involve monodispersed NPs [46]; 2) upon deposition into the lung, particles will interact with lung lining fluid and become coated with proteins and other biomolecules; 3) regardless of the suspension coating or dispersion at the time of exposure, particles may agglomerate within the lung upon deposition [47]; 4) we wanted to keep the material as pristine as possible for better comparison to the uncoated, pristine material used for inhalation; and, 5) in a pilot study, we determined that pretreatments with coating or sonication can modify the inflammatory response (Additional file 1: Figure S1). We found that pretreatment with dispersion medium (DM; 1,2-dipalmitoyl-sn-glycero-3-phosphocholine + albumin in saline [48]) resulted in significantly lower neutrophil influx than with saline alone (Additional file 1: Figure S1A). These findings are consistent with a study by Morimoto et al. [49] where fullerenes prepared with a 0.1 mg/mL coating of Tween-80 were not able to induce inflammatory effects when delivered by either whole body inhalation or intratracheal instillation. In addition, we observed that increased sonication time led to a significant decrease in neutrophil influx (Additional file 1: Figure S1B). In order to detect quantifiable differences between instilled and inhaled animals for our study, we kept the material as pristine as possible by suspending the material in saline and using only a 5 sec sonication time. Nonetheless, our findings regarding the impact of dispersant and sonication time on acute inflammation provide additional caveats when performing and interpreting results from studies that employ bolus delivery of NPs.

#### ***Inflammatory cell influx and BALF biochemical parameters following single high dose TiO_2_ NP exposure at high and low deposited dose rates***

We performed single high dose exposures by whole body inhalation or intratracheal instillation and collected BALF to assess inflammatory response induction and resolution over a 7 day period (Figure 2 and Table 3). The response endpoints are plotted in Figure 2 as a percentage of corresponding control in order to clearly depict the differences in response over time between the two exposure methods. All raw data appear in Table 3. After an initial decrease, BALF cell numbers increased significantly 24 hr after intratracheal instillation with TiO_2_; this effect was also significantly higher than the corresponding inhalation exposure group (Figure 2A and Table 3). The cell number changes were resolved within 7 days post exposure, despite the fact that TiO_2_ was still present in the lung. There were no time dependent changes in cell number following TiO_2_ inhalation.

**Table 3:** **BALF cellular and biochemical parameters following single exposures to TiO**_**2 **_**NPs at different dose rates**

**A)**	**Whole body inhalation**					
	**Air Controls**	**TiO**_**2**_				
**Post exposure time**		**4 hr**	**8 hr**	**24 hr low dose**	**24 hr high dose**	**7 days**
**Cell viability (%)**	95.6 ± 1.2	95.1 ± 0.7	94.9 ± 0.9	94.7 ± 1.0	92.5 ± 2.3	95.5 ± 1.4
**Total cells (Number × 10**^**7**^**)**	1.39 ± 0.21	1.53 ± 0.07	1.41 ± 0.10	1.62 ± 0.14*	1.32 ± 0.07	1.41 ± 0.16
**Alveolar macrophages (%)**	99.2 ± 0.3	98.7 ± 1.1	98.6 ± 0.7	97.6 ± 0.9	92.7 ± 1.4	99.0 ± 0.5
**(Number × 10**^**7**^**)**	(1.36 ± 0.2)	(1.52 ± 0.07)	(1.39 ± 0.11)	(1.58 ± 0.12)	(1.22 ± 0.07)	(1.40 ± 0.16)
**Neutrophils (%)**	0.5 ± 0.1	0.7 ± 0.9	0.8 ± 0.6	1.6 ± 0.6	6.2 ± 1.5	0.6 ± 0.4
**(Number × 10**^**5**^**)**	(1.26 ± 0.69)	(1.01 ± 1.41)	(1.14 ± 0.70)	(2.59 ± 1.08)	(8.18 ± 1.86)*	(0.89 ± 0.56)
**Lymphocytes (%)**^**a**^	0.3 ± 0.3	0.6 ± 0.3	0.4 ± 0.3	0.8 ± 0.4	1.1 ± 0.2	0.6 ± 0.4
**(Number × 10**^**4**^**)**^**b**^	(3.92 ± 3.31)	(10.12 ± 14.05)	(5.41 ± 4.91)	(13.92 ± 7.67)	(14.89 ± 2.34)	(8.45 ± 4.99)
**LDH release (nmol/min/mL)**	39.82 ± 3.98	49.31 ± 4.91*	55.01 ± 3.18*	59.91 ± 19.37	38.93 ± 4.99	42.31 ± 2.37
**β-Glucuronidase activity (nmol/min/mL)**^**c**^	0.18 ± 0.03	0.08 ± 0.03*	0.11 ± 0.03*	0.37 ± 0.11*	0.20 ± 0.02	.20 ± 0.03
**BALF Protein (mg/mL)**^**c**^	0.13 ± 0.01	0.14 ± 0.02	0.13 ± 0.02	0.08 ± 0.02*	0.15 ± 0.01	0.12 ± 0.04
**B)**	**Intratracheal instillation**					
	**Saline Controls**	**TiO**_**2**_				
**Post exposure time**		**4 hr**	**8 hr**	**24 hr low dose**	**24 hr high dose**	**7 days**
**Cell viability (%)**	95.6 ± 1.1	96.0 ± 0.3	94.7 ± 1.3	94.1 ± 0.8	94.5 ± 1.7	93.7 ± 0.7
**Total cells (Number × 10**^**7**^**)**	1.52 ± 0.18	1.39 ± 0.14	1.40 ± 0.32*	1.66 ± 0.08	2.09 ± 0.11*Φ	1.50 ± 0.06
**Alveolar macrophages (%)**	98.4 ± 0.3	95.0 ± 2.6	89.4 ± 6.9*Φ	95.5 ± 1.4	67.7 ± 3.1*Φ	96.6 ± 1.3
**(Number 10**^**7**^**)**	(1.54 ± 0.12)	(1.32 ± 0.11)	(1.23 ± 0.17)*	(1.59 ± 0.09)	(1.41 ± 0.08)	(1.45 ± 0.04)
**Neutrophils (%)**	1.0 ± 0.2	3.8 ± 2.8*Φ	9.8 ± 7.1*Φ	3.7 ±1.4*	31.1 ± 2.6*Φ	1.9 ± 1.7
**(Number × 10**^**5**^**)**	(1.47 ± 0.43)	(5.52 ± 4.35)*Φ	(15.46 ± 15.42)*Φ	(6.10 ± 2.31)*Φ	(65.02 ± 6.56)*Φ	(2.84 ± 2.66)
**Lymphocytes (%)**^**a**^	0.7 ± 0.3	0.7 ± 0.2	0.8 ± 0.3	0.8 ± 0.1	1.2 ± 0.8	0.9 ± 0.2
**(Number × 10**^**4**^**)**^**b**^	(9.55 ± 2.68)	(9.95 ± 3.03)	(10.33 ± 3.76)	(13.74 ± 1.18)	(25.00 ± 18.38)	(13.19 ± 3.34)
**LDH release (nmol/min/mL)**	46.32 ± 7.36	47.99 ± 3.78	55.19 ± 5.76	68.01 ± 9.12*	76.59 ± 4.15*Φ	58.60 ± 4.22*Φ
**β-Glucuronidase activity (nmol/min/mL)**^**c**^	0.22 ± 0.03	0.10 ± 0.03*	0.15 ± 0.02*	0.30 ± 0.06	0.52 ± 0.07*Φ	0.30 ± 0.05*Φ
**BALF protein (mg/mL)**^**c**^	0.12 ± 0.02	0.16 ± 0.02	0.18 ± 0.01*Φ	0.07 ± 0.00*	0.14 ± 0.02	0.11 ± 0.01

Values are group means (n = 5) ± SD for inhalation (4 hr duration) and instillation exposures at 4, 8, 24 hr and 7 days post beginning of exposure. *, significantly different from corresponding controls; Φ, significantly different from whole body inhalation at the same post-exposure time point; ^a^, main effects of both time and exposure method; ^b^, main effect of exposure method (p < 0.05); ^c^, one outlier was identified through analysis of the residuals and removed.

Overall, there were no statistically significant elevations in macrophages that would explain the observed changes in total cell numbers (Figure 2B and Table 3). There was a transient decrease following instillation exposure that could have been due to adhesive changes. Not surprisingly, there were also no changes in the number (or percentage) of lymphocytes in BALF (Table 3). The change in total cell number, thus, was primarily due to neutrophil influx (Figure 2C and Table 3). We observed significant increases from saline controls at 4, 8 and 24 hr post TiO_2_ instillation and a small transient increase in neutrophil number 24 hr after inhalation exposure. The magnitude of the neutrophil response following instillation is more than 4 times higher compared to inhalation exposure when the response is at its peak. By 7 days post exposure, the inflammatory cell changes had completely resolved regardless of exposure method. Our findings of peak inflammation occurring 24 hr after exposure are consistent with historical data from our laboratory and other published findings [34,50-53].

We also evaluated LDH release and β-glucuronidase activities (Figure 2D & E) in order to determine if deposited dose rate influences the cell membrane integrity or lysosomal membrane integrity, respectively. We observed that these response patterns followed similar trends as the cellular data, as expected, except that the instillation response did not fully resolve within 7 days. The apparently overlapping symbols in Figure 2E are a result of depicting the data as percent of control, meaning the saline and air controls have slightly different baseline values. Despite the small increases in LDH and β-glucuronidase activities, there was no change in lavage cell viability (Table 3) following exposure to TiO_2_ by either exposure method. Furthermore, BALF protein levels, indicative of epithelial barrier permeability changes (Table 3), were only transiently increased 8 hr after instillation exposure. These findings confirm previous reports that TiO_2_ is not directly cytotoxic, especially with a lung deposited dose in the microgram range [15,54-57].

#### ***Inflammatory cell influx and BALF biochemical parameters following single low dose TiO_2_ NP exposure at high and low deposited dose rates***

**Figure 3:**
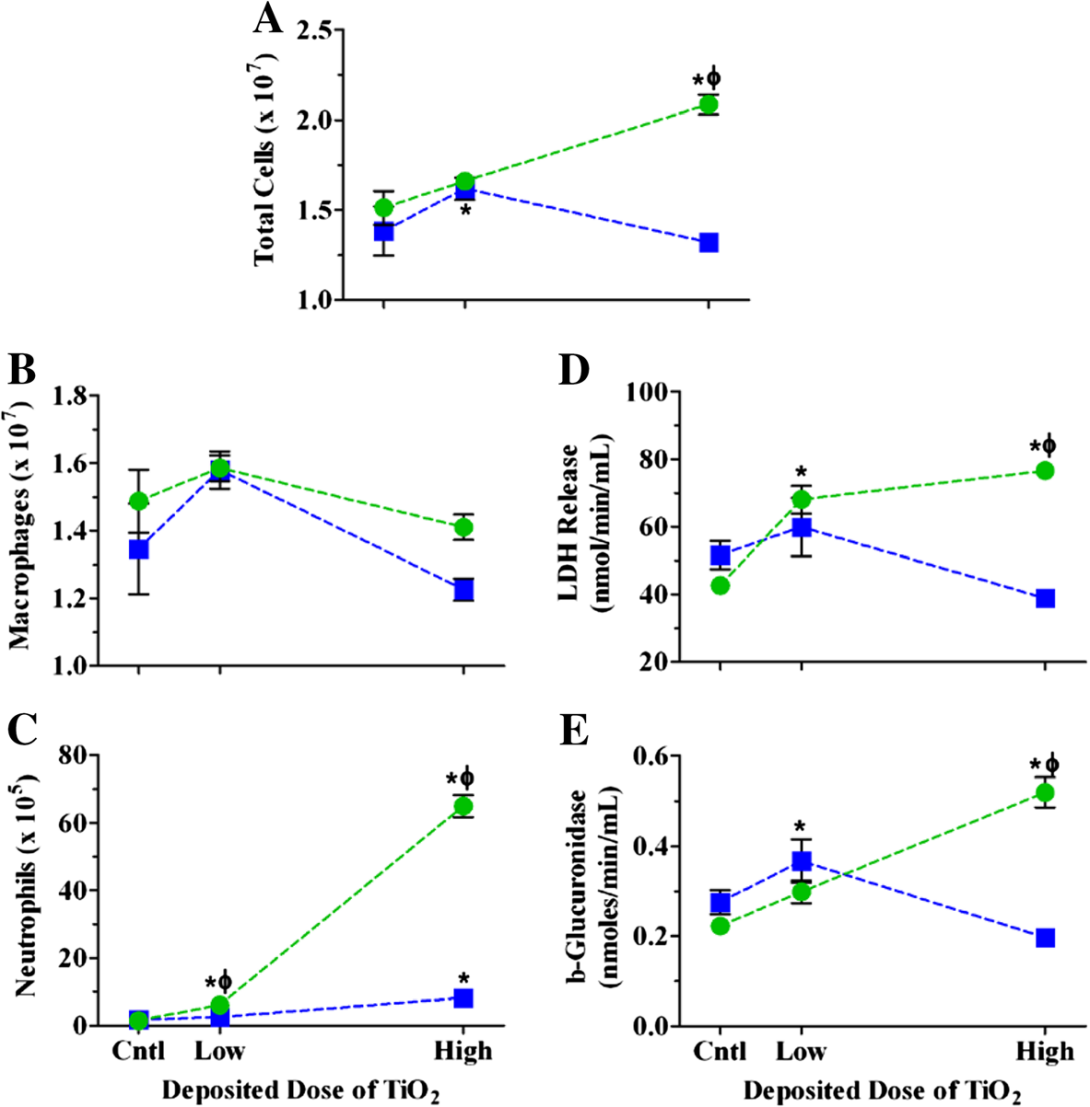
**Dose response relationships for BALF cellular and biochemical parameters 24 hr post exposure to TiO**_**2 **_**NPs.** The numbers of cells **(A)**, macrophages **(B)**, and neutrophils **(C)**, and LDH **(D)** and β-glucuronidase activities **(E)** 24 hr post inhalation (*blue squares*) or instillation (*green circles*) exposure to increasing deposited doses of TiO_2_ NPs. Values are group means ± SE (n = 5) and are represented as a percentage of respective controls. *, significantly different from corresponding controls; Φ, significantly different between exposure methods (p < 0.05).

By incorporating a lower deposited dose into the study design, we were able to better characterize the impact of dose rate on responses to TiO_2_ NPs. We chose to evaluate responses only 24 hr after exposure, as this is when the most robust inflammatory responses were found at the higher deposited dose. We observed dose dependent increases in total cell number and neutrophil influx and only transient increases in macrophages following instillation (Figure 3 and Table 3). After inhalation, there was a significant increase in cell number at the low dose, due to small increases in macrophages and neutrophils. Although inhalation exposure did result in dose dependent increases in neutrophils, these were significantly lower in magnitude as compared to instillation at the lower deposited dose (Figure 3C and Table 3). LDH and β-glucuronidase increased in a dose dependent manner following instillation, with only transient increases at the lower dose following inhalation (Figures 3D and E, Table 3). Overall, however, these data show more robust responses by instillation than inhalation at both doses, confirming that dose rate plays a role in the inflammatory response at lower deposited doses as well as higher doses.

#### ***Inflammatory mediator release and oxidative stress levels following single TiO_2_ NP exposures at high and low deposited dose rates***

**Figure 4:**
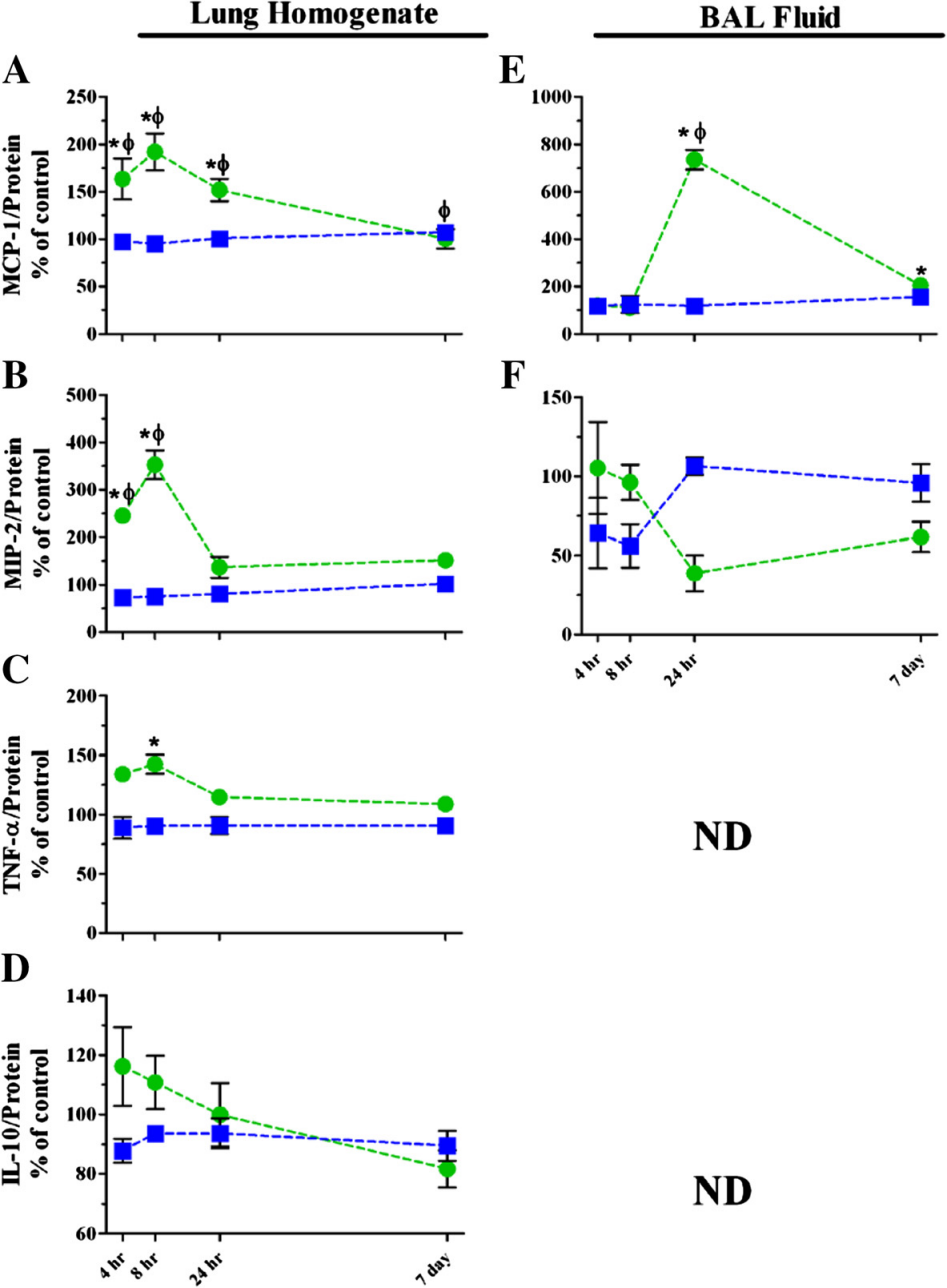
**Inflammatory mediator release in lung homogenates and BALF supernatants following single high dose exposure to TiO**_**2 **_**NPs.** Changes in MCP-1 (**A***, homogenates;***E***, BALF supernatants*), MIP-2 (**B***, homogenates;***F***, BALF supernatants*), TNF-α (**C***, homogenates*) and IL-10, (**D***, homogenates*) following inhalation (*blue squares*) or instillation (*green circles*) are graphed as percentage of controls. Values are group means ± SE (n = 5). ND, levels were below the limit of detection by ELISA (TNF-α and IL-10 ~15 pg/mL). *, significantly different from corresponding controls; Φ, significantly different between exposure methods (p < 0.05).

We measured several inflammatory mediators by ELISA in order to identify the drivers of the neutrophil responses following instillation and inhalation exposures to TiO_2_ and to determine if the mechanisms of response are different based on dose rate. Several studies have demonstrated links between NP exposure, oxidative stress and pro-inflammatory mediator release [51,58,59]. We selected mediators that are well-documented in the nanotoxicology literature as playing a role in RT inflammation, namely: monocyte chemoattractant protein-1 (MCP-1), macrophage inflammatory protein-2 (MIP-2), tumor necrosis factor alpha (TNF-α) and interleukin-10 (IL-10) (Figure 4 and Table 4). For example, MCP-1 has been shown to induce neutrophil chemotaxis to the lungs in response to inflammatory stimuli [60]. Similarly, MIP-2, the rat homologue of human IL-8, has been implicated in the aforementioned studies as a key mediator in the inflammatory response and is released by several cell types, including type II alveolar epithelial cells and alveolar macrophages. Another study suggested that TNF-α was involved with the stimulation of MIP-2 mRNA transcription for enhanced neutrophil chemotaxis [61]. Regarding TiO_2_ NP exposure and the induction of the inflammatory response, specifically, 1–10 nm (0–800 μg/mL) nanosized TiO_2_ was found to induce the production of IL-8 by freshly isolated human PMNs [62]. In addition, IL-10 levels were increased along with the total number of neutrophils in the lungs of rats for 1–2 days after instillation exposure to 1, 5 and 7.5 mg/kg TiO_2_, indicating increases in inflammation, which resolved within 16 days of exposure [63].

**Table 4:** **Inflammatory mediator and oxidative stress release in BALF and lung homogenates following TiO**_**2 **_**NP exposure at different dose rates**

**A)**	**Whole body inhalation**					
	**Air Controls**	**TiO**_**2**_				
**Post exposure time**		**4 hr**	**8 hr**	**24 hr low dose**	**24 hr high dose**	**7 days**
**BALF MCP-1 (pg/mg protein)**	5878 ± 1292	6879 ± 3514	7285 ± 4762	17620 ± 3629*	6936 ± 1590	9112 ± 1977
**BALF MIP-2 (pg/mg protein)**	1592 ± 456	1022 ± 787	893 ± 491	957 ± 343	1696 ± 194	1526 ± 424
**Homogenate MCP-1 (pg/mg protein)**	578 ± 52	564 ± 64	551 ± 18	527 ± 68	582 ± 72	621 ± 39
**Homogenate MIP-2 (pg/mg protein)**	18.85 ± 3.84	13.76 ± 1.36	14.21 ± 1.88	18.59 ± 3.67	15.23 ± 4.02	19.29 ± 2.91
**Homogenate TNF-α (pg/mg protein)**	4.66 ± 0.71	4.14 ± 0.95	4.21 ± 0.42	4.79 ± 0.56	4.23 ± 0.75	4.22 ± 0.32
**Homogenate IL-10 (pg/mg protein)**^**a**^	10.85 ± 0.74	9.53 ± 0.99	10.16 ± 0.60	10.12 ± 1.14	10.16 ± 1.22	9.71 ± 1.20
**Homogenate HO-1 (ng/mg protein)**	1.62 ± 0.15	1.45 ± 0.10	1.69 ± 0.41	1.56 ± 0.19	1.74 ± 0.18	1.60 ± 0.25
**BAL pellet HO-1 (ng/mg protein)**^**b**^	101 ± 14	91 ± 53	118 ± 28	104 ± 9	134 ± 13	121 ± 11
**B)**	**Intratracheal instillation**					
	**Saline Controls**	**TiO**_**2**_				
**Post exposure time**		**4 hr**	**8 hr**	**24 hr low dose**	**24 hr high dose**	**7 days**
**BALF MCP-1 (pg/mg protein)**	9026 ± 2037	10770 ± 5619	12590 ± 3402	19340 ± 1913.70*	66450 ± 8421*Φ	18430.00 ± 3593*
**BALF MIP-2 (pg/mg protein)**	2008 ± 2486	2114 ± 1309	1932 ± 501	1413 ± 412	779 ± 508	1240 ± 428
**Homogenate MCP-1 (pg/mg protein)**	1084 ± 231Φ	1774 ± 525*Φ	2084 ± 473*Φ	429 ± 40*	1647 ± 287*Φ	1087 ± 249Φ
**Homogenate MIP-2 (pg/mg protein)**	8.64 ± 2.71Φ	21.23 ± 2.68*Φ	30.53 ± 5.86*Φ	6.78 ± 0.64*Φ	11.87 ± 4.29	13.12 ± 1.97
**Homogenate TNF-α (pg/mg protein)**	3.86 ± 0.89	5.19 ± 0.54	5.51 ± 0.68*	3.76 ± 0.59	4.44 ± 0.53	4.21 ± 0.37
**Homogenate IL-10 (pg/mg protein)**^**a**^	8.83 ± 2.12	10.26 ± 2.61	9.79 ± 1.77	7.27 ± 1.41	8.82 ± 2.10	7.22 ± 1.22
**Homogenate HO-1 (ng/mg protein)**	1.80 ± 0.58	2.79 ± 0.42*Φ	2.56 ± 0.17*Φ	1.27 ± 0.08*	3.38 ± 0.49*Φ	1.51 ± 0.34
**BAL pellet HO-1 (ng/mg protein)**^**b**^	101 ± 16	96 ± 6	124 ± 15	114 ± 17	135 ± 31	118 ± 6

Values are group means (n = 5) ± SD for inhalation (4 hr duration) and instillation exposures at 4, 8, 24 hr and 7 days post beginning of exposure. *, significantly different from corresponding controls; Φ, significantly different from whole body inhalation at the same post-exposure time point; ^a^, significant main effect of exposure method; ^b^, significant main effect of time (p < 0.05).

In the present studies, intratracheal instillation resulted in significant increases in MCP-1 in both homogenized lung tissue and BALF (Figure 4A and E, Table 4). There was, however, no significant change in MCP-1 in the animals exposed to TiO_2_ NPs by inhalation, suggesting that it did not play a significant role in the small but statistically significant neutrophil influx that was observed (Figures 2 and 3; Table 3). MCP-1 was the only inflammatory mediator that we found to have higher concentrations in the BALF than the lung homogenates. This is likely due to MCP-1 being released by the neutrophils themselves following recruitment into the lung, thus propagating the inflammatory response [64]. Also, MCP-1 was the only significantly increased inflammatory mediator following low dose single exposure to TiO_2_ NPs (Table 4). MIP-2 showed statistically significant increases from controls in lung homogenates following high dose instillation, and this response was also significantly higher than the MIP-2 released following inhalation in the early phase of the inflammatory response (Figure 4B and Table 4). In BALF, the MIP-2 response was more variable (Figure 4F and Table 4) and, while the general trends over time were different between the exposure methods, there were no statistically significant changes. TNF-α had similar trends to the MIP-2 release in the homogenates, but the overall response was lower (Figure 4C and Table 4). TNF-α was not detectable in BALF. We also evaluated the release of an anti-inflammatory cytokine, IL-10, in order to characterize the resolution of the response (Figure 4D and Table 4), and observed only a main effect of exposure method. Based on our findings regarding patterns of release of MCP-1, MIP-2, TNF-α and IL-10, we conclude that these mediators played a role in driving the inflammatory response to TiO_2_ NPs that are delivered via instillation, but not inhalation. Certainly, there are other mediators that can be investigated to further characterize the differences in response by both exposure methods. Thus, we have built upon the findings of Slikker et al. [13] – that the dose determines the mechanism – by suggesting that the mechanisms of the inflammatory response to TiO_2_ are inherently different when it is deposited at different dose rates.

**Figure 5:**
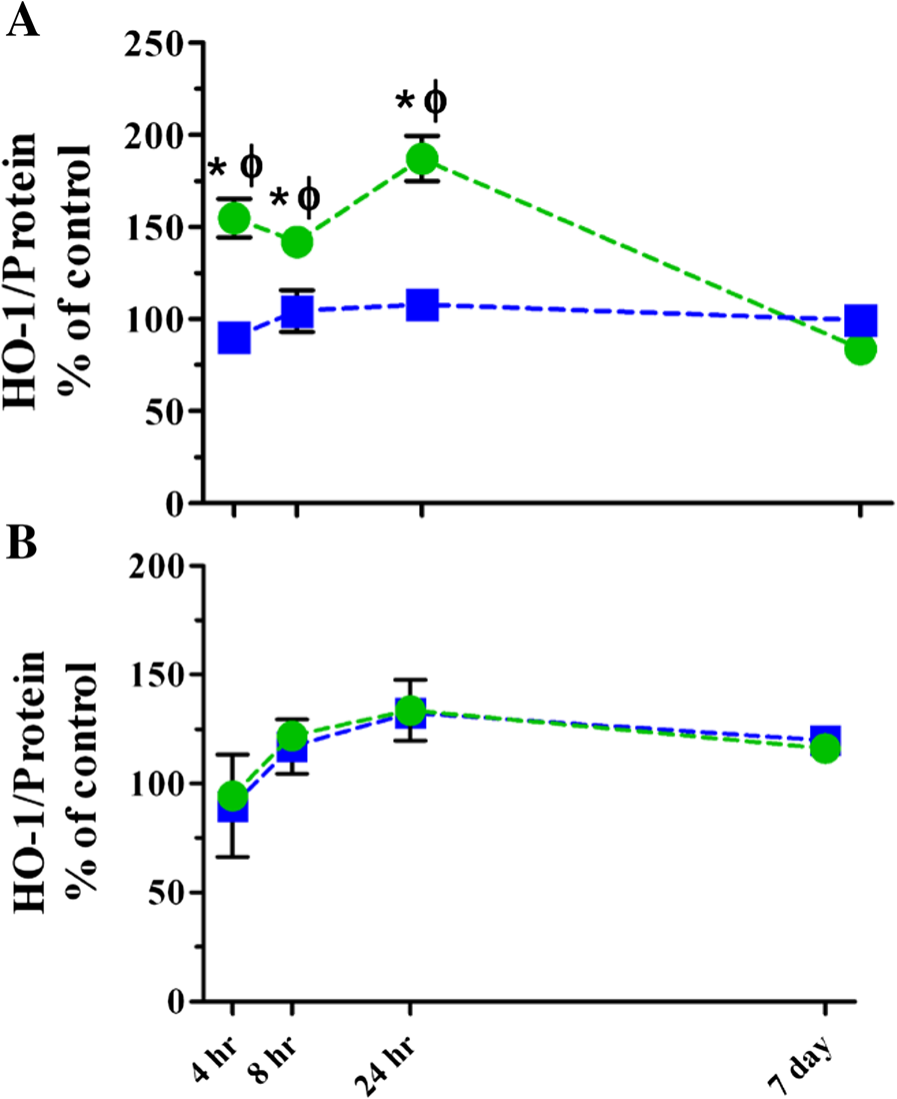
**HO-1 levels in lung homogenates and BALF cell pellets following single high dose exposure to TiO**_**2 **_**NPs.** HO-1 levels in lung homogenates **(A)** and BALF cell pellets **(B)** following inhalation (*blue squares*) or instillation (*green circles*) exposure. Values are group means ± SE (n = 5) and represented as percentage of controls. *, significantly different from corresponding controls; Φ, significantly different between exposure methods (p < 0.05).

We also evaluated changes in the early oxidative stress marker, heme oxygenase-1 (HO-1; Figure 5 and Table 4), which has been shown to increase in target cells that are exposed to NPs *in vitro*[65]. Previous studies have shown that type II cells and alveolar macrophages produce HO-1 in response to TNF-α release [66,67]. Here, we saw significant increases in lung homogenate HO-1 following instillation of TiO_2_ as compared to corresponding controls and with respect to animals that were exposed by inhalation at 4, 8 and 24 hr post exposure (Figure 5A and Table 4). Interestingly, the HO-1 levels in BALF cell pellets only modestly increased over time and the trend was similar for either method, indicating that its production by lung inflammatory cells was not affected by the deposited dose rate like it was in the lung tissue (Figure 5B and Table 4).

### Lung inflammatory responses following repeated exposures to a high dose of TiO_2_ NPs

**Figure 6:**
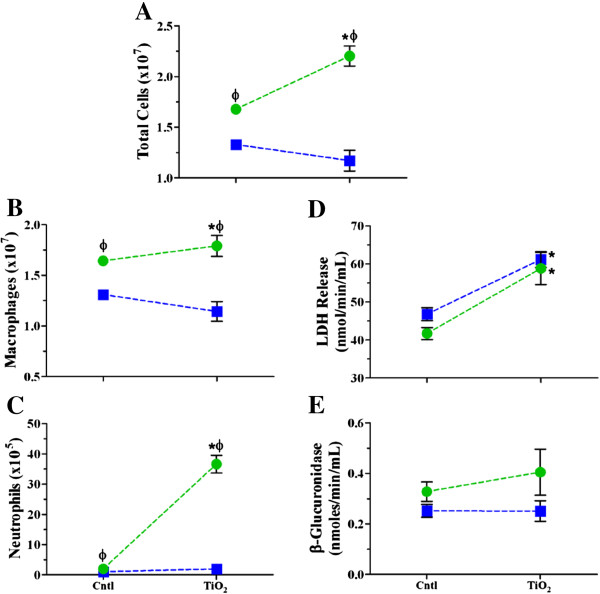
**BALF cellular and biochemical parameters following repeated high dose exposure to TiO**_**2 **_**NPs.** The numbers of cells **(A)**, macrophages **(B)**, and neutrophils **(C)**, and LDH **(D)** and β-glucuronidase activities **(E)** were assessed 24 hr post inhalation (*blue squares*) or instillation (*green circles*) exposure. Values are group means ± SE (n = 5). *, significantly different from corresponding controls; Φ, significantly different between exposure methods (p < 0.05).

In order to further characterize the effects of dose rate, we fractioned the deposited dose over 4 consecutive days of exposures either by intratracheal instillation or whole body inhalation. This exposure scenario effectively changed the rate at which the total mass of TiO_2_ was deposited into the lung to much lower rates than with single exposures (0.21 μg/min, repeated inhalation; 0.71 μg/min, single inhalation; 5400 μg/min, repeated instillation; 200,000 μg/min single instillation). The total deposited amounts are shown in Table 2 and, again, were not statistically significantly different between exposure methods. The total number of cells, macrophages and neutrophils were all significantly increased from control values following instillation and were also significantly greater than responses observed following TiO_2_ inhalation (Figure 6 and Table 5). LDH release was significantly increased by repeated inhalation and instillation exposures (Figure 6D); no significant changes were detected for β-glucuronidase activity (Figure 6E). Similar to the single exposure scenario, there were also no statistically significantly different changes observed in cell viability following repeated exposure (Table 5). Statistically significant differences from control or between exposure methods were not observed for BALF protein, only a statistically significant main effect of TiO_2_ exposure (Table 5). Overall, the effects of deposited dose rate for the repeated exposure model were similar to what was observed following single exposures, except that repeated exposure apparently dampened the inflammatory response. This may be due to animals undergoing adaptation as has been observed with other inflammatory stimuli [68,69]. Also, the number and percentages of lymphocytes were significantly increased following repeated instillation exposure, which is also an indicator of adaptation [70]. Indeed, the repeated exposure model confirms the importance of dose rate when designing experiments to characterize risks associated with NPs in humans.

**Table 5:** **BALF cellular and biochemical parameters following repeated exposures to TiO**_**2 **_**NPs at different dose rates**

	**Whole body inhalation**		**Intratracheal instillation**	
	**Air**	**TiO**_**2**_	**Saline**	**TiO**_**2**_
**Cell viability (%)**	95.1 ± 1.4	95.1 ± 2.5	95.0 ± 2.3	94.2 ± 0.8
**Total cells (Number ×10**^**7**^**)**	1.33 ± 0.10	1.17 ± 0.23	1.68 ± 0.06Φ	2.20 ± 0.22*Φ
**Alveolar macrophages (%)**	98.7 ± 0.4	97.7 ± 0.5	97.9 ± 0.6	81.2 ± 3.8*Φ
**(Number ×10**^**7**^**)**	1.31 ± 0.10	1.14 ± 0.22	1.64 ± 0.06Φ	1.79 ± 0.23
**Neutrophils (%)**	0.8 ± 0.2	1.6 ± 0.6*	1.2 ± 0.3	16.8 ± 3.6*Φ
**(Number ×10**^**5**^**)**	1.02 ± 0.36	1.96 ± 1.00	2.00 ± 0.60Φ	36.59 ± 6.60*Φ
**Lymphocytes (%)**	0.6 ± 0.2	0.7 ± 0.2	0.8 ± 0.5	2.1 ± 0.5*Φ
**(Number ×10**^**4**^**)**	7.62 ± 2.25	7.53 ± 2.45	14.05 ± 7.29	45.99 ± 11.86*Φ
**LDH release (nmol/min/mL)**	46.83 ± 3.72	61.19 ± 4.62*	41.72 ± 3.48	58.81 ± 9.43*
**β-Glucuronidase activity (nmol/min/mL)**	0.25 ± 0.06	0.25 ± 0.09	0.33 ± 0.09	0.40 ± 0.20
**BALF protein (mg/mL)**^**a**^	0.12 ± 0.01	0.15 ± 0.02	0.12 ± 0.01	0.13 ± 0.01

Values are group means (n = 5) ± SD for inhalation (4 hr duration) and instillation exposures at 24 hr post beginning of exposure. *, significantly different from corresponding controls; Φ, significantly different from whole body inhalation at the same post-exposure time point; ^a^, significant main effect of TiO_2_ exposure (p < 0.05).

### Summary of the role of deposited TiO_2_ dose rate

**Figure 7:**
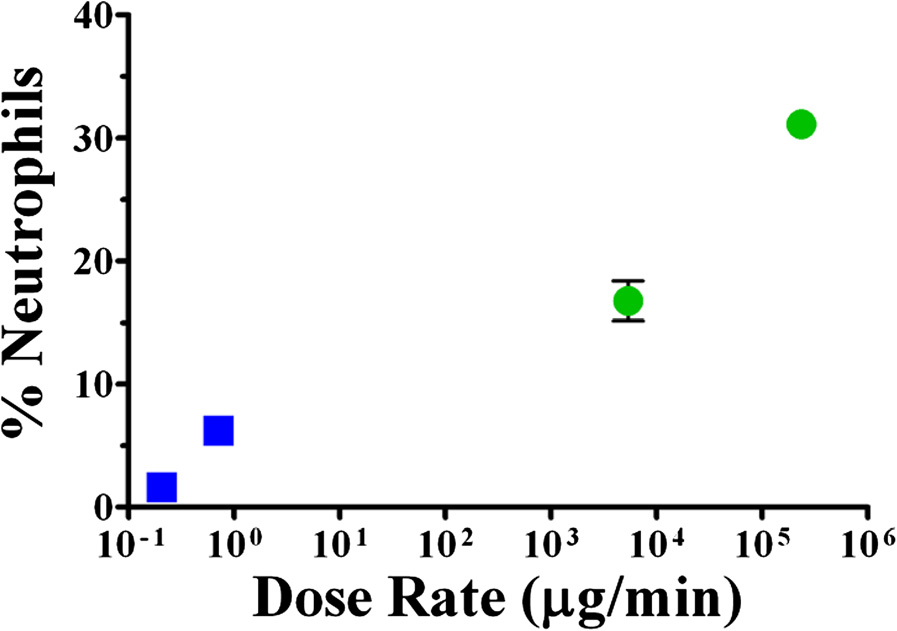
**Percentage of neutrophils 24 hr after single and repeated exposure to high dose TiO**_**2 **_**at different dose rates.** The percentage of neutrophils in BALF at 24 hr post inhalation (*blue squares*) or instillation (*green circles*) exposure plotted according to deposited dose rate (~0.21, repeated inhalation; ~0.71, high dose single inhalation; ~5,400, repeated instillation; and ~240,000 μg/min, high dose single instillation). Symbols are group means ± SE (n = 5).

The role of dose rate based on neutrophil influx following both repeated and single high dose exposures is represented in Figure 7, with response plotted as a function of the deposited dose rate. Clearly, as deposited dose rate increases, there is a corresponding increase in the neutrophil response. TiO_2_ instillation not only resulted in significantly more robust neutrophil influx, but also increases in LDH and β-glucuronidase activities, and MCP-1, MIP-2 and TNF-α inflammatory mediator release in lung homogenates when compared to controls and to TiO_2_ inhalation. MCP-1 in BALF supernatants and HO-1 levels in lung homogenates were also significantly increased from controls and compared to inhalation. Although there are several other inflammatory mediators and oxidative stress endpoints that could be investigated, the present results demonstrate that the high deposited dose rate exposures elicited higher inflammation due to signaling from the lung parenchyma as well as resident and infiltrating inflammatory cells. Whole body inhalation exposure to TiO_2_ NPs resulted in a low level, but statistically significant neutrophil response that was evident at 24 hr post exposure when compared to air exposed animals, with few statistically significant differences in the other response endpoints. These findings suggest that the inflammatory response following low dose rate inhalation exposure was initiated by resident inflammatory cells in the lung. Regardless of the exposure method, the inflammatory response had largely subsided by 7 days post exposure, despite the fact that TiO_2_ NPs still remained in the lungs.

Our findings regarding the role of dose rate in TiO_2_ NP induced inflammatory response outcomes may only be relevant for other poorly soluble NPs. Indeed, when the same deposited dose of oil combustion particles, which are more reactive than TiO_2_ and a mixture of metal oxides, were delivered by intratracheal instillation or nose-only inhalation, the neutrophil, BALF protein, lung histopathology and airway responsiveness endpoints were similar between the two methods [71]. Therefore, further investigations to elucidate the mechanisms of response following different dose rate deliveries are needed for more reactive metal and metal oxide NPs. Post exposure (60–90 days) evaluation of the lung tissue histopathology would also provide further characterization of the resolution of the acute inflammatory response.

## Conclusions

The deposited dose rate determines the mechanism and magnitude of the acute inflammatory response in the RT when the ILBs are the same. Our data suggest that results from intratracheal instillation exposure studies overestimate acute TiO_2_ NP toxicity and that careful consideration should be given to dose rate in the context of risk characterization. However, intratracheal instillation could still be appropriate for hazard ranking (i.e., comparing one nanomaterial to another). We presume that our conclusion that increased dose rate results in increased response outcomes is not specific to TiO_2_ NPs and that this phenomenon would most likely extend to other poorly soluble particles with low cytotoxicity, regardless of their size.

## Methods

### Animals

Specific pathogen-free male Fischer 344 rats (Harlan; Frederick, MD; 175–200 g body weight) were housed in filter-top plastic cages and given free access to food (5001; Purina Mills, LLC, St. Louis, MO) and water in a humidity and temperature controlled room with a 12 hr light–dark cycle. Prior to use in experimental protocols, all animals were acclimated for at least 1 week in an Association for Assessment and Accreditation of Laboratory Animal Care International-accredited facility. Animals were treated humanely and with regard to the alleviation of suffering in accordance with a protocol that was approved by the University of Rochester’s Committee on Animal Resources. Animals were randomly distributed among the treatment groups, with 3–5 rats per group for dosimetry and separate groups of 5 rats for assessing acute inflammatory parameters.

### Achieving similar deposited doses of TiO_2_

A high deposited dose of ~200 μg TiO_2_ was selected so that we could compare our findings with existing data sets generated in our own laboratory and other literature reports [34,52,72,73]. The lower dose of ~45 μg was chosen after performing preliminary experiments that interrogated the dose response curve for neutrophil influx 24 hr after intratracheal instillation, in which we began observing significant inflammatory effects compared to saline controls. While it is straightforward to achieve the target doses for instillation exposures, lower RT deposition fractions derived from the Multiple Particle Path Dosimetry (MPPD) model [74] were used to calculate aerosol concentrations that would produce the desired doses upon inhalation exposure. In addition, the deposited doses (ILBs) following inhalation and intratracheal instillation exposures were verified through quantification of Ti content in lung tissue (*see below*).

### Whole body inhalation exposures

Whole body inhalation was used as a low dose rate delivery method. Other exposure methods, such as nose-only inhalation, can minimize the deposition of NPs on the animals’ skin and fur and thus reduce oral uptake of NPs. However, a significant disadvantage to such methods is that the animals are subjected to higher levels of stress and would not be acclimated to exposure conditions following a single or even repeated exposure for only 4 days [75]. Due to the uncertain contribution of this stress to response outcomes, we decided to compare our intratracheal instillation response outcomes to those following whole body inhalation NP exposure. Furthermore, much of the historical literature to which we wished to compare our results describes effects of TiO_2_ NPs that were delivered via whole body inhalation exposures.

Rats were randomly placed in a 60 L compartmentalized, polycarbonate (Lexan) chamber, which was under slight negative pressure with an internal horizontal flow of 35 L/min. TiO_2_ powder (AEROXIDE P25 powder, Evonik, Germany; primary particle size, 25 nm; 80% anatase, 20% rutile crystal phase; BET surface area 57 m^2^/g, [4]) was fed by a screw mechanism into a jet mill (JET-O-MIZER™, Fluid Energy Equipment Division, Telford, PA), producing aerosol concentrations of either 33 ± 4 mg/m^3^ or 13 ± 1 mg/m^3^ (for 4 hr). The higher aerosol concentration of 33 ± 4 mg/m^3^ was used to achieve the higher deposited dose for the single exposure. The 13 ± 1 mg/m^3^ aerosol concentration was used for the single, low deposited dose and also for the repeated exposure for 4 hr over 4 consecutive days. Control animals were exposed to filtered air. The TiO_2_ aerosol mass concentrations were determined gravimetrically via filter (PALLFLEX® Emfab; PALL Life Sciences, Port Washington, NY) samples drawn from the chamber that were collected every 15 min. The MMAD was determined by an eight-stage Nano-MOUDI impactor (MSP Corp., Shoreview, MN) and the CMD by a model 1000XP wide range particle spectrometer (MSP Corp.). The size characteristics of the TiO_2_ aerosols were similar for the single and repeated exposure concentrations (Table 1).

### Intratracheal instillation exposures

TiO_2_ NPs were cup horn sonicated (Sonics VCX 750 Vibra Cell, Sonics and Materials, Inc., Newtown, CT) for 5 sec at 29% amplitude in 0.9% sterile saline and vortexed for ~30 sec immediately prior to intratracheal instillation. For the experiments in Additional file 1: Figure S1, the NPs were sonicated for 5 sec, 5 min or 30 min in either dispersion medium or saline. Rats were anesthetized with 4.5% isoflurane until their breathing was slow and shallow, after which the rats were placed in a supine position with the head elevated. A modified pediatric otoscope was used to visualize the vocal cords. A 20 gauge, 1.5 inch, Teflon® catheter sheath was inserted through the vocal cords until the tip was ~3-5 mm above the bronchial carina. In synchrony with the inspiratory phase of the breathing cycle, 250 μL of the TiO_2_ suspension was instilled into the lung. Controls were exposed to saline that was sonicated as described above. Mock intratracheal instillations directly into platinum crucibles showed that no significant losses of TiO_2_ occurred in the instillation needle or syringe when compared to the deposited doses in the lungs.

The hydrodynamic size of a 1:10 dilution of the 800 μg/mL suspension of TiO_2_ for intratracheal instillation was determined by DLS (Nano ZS Zetasizer, Malvern Instruments, Westborough, MA) and LDS (Partica LA-950 V2; Horiba Instruments, Inc., CA) as previously described [18]. The measurements by DLS and LDS were taken after the material was sonicated for ~5 sec and then vortexed for ~30 sec, every 15 minutes for 1 hr, mimicking the preparation used prior to instilling the material and accounting for the time it takes to instill all of the rats in one group (~1 hr).

### Dissolution of TiO_2_

The dissolution rate of TiO_2_ NPs was determined with a dynamic flow-through system as previously described [76]. Briefly, NPs (~0.8 mg) were suspended in 1 mL of dissolution buffer before being injected into the upper chamber of a dialysis cell fitted with a 3,500 molecular weight cellulose ester asymmetric membrane (Spectra/Pore®, Gardena, CA; effective pore size ~3.5 nm). The Ti-free dissolution buffers simulated extracellular lung lining fluid (pH = 7.4) and intraphagolysosomal fluid (pH = 4.5), respectively. The buffers flowed into the dialysis cells at a rate of 60 μL/min, or ~3 mL/hr; the outlet ports of the dialysis cells were connected to a fraction collector. The dialysis cells were submerged in a 37°C water bath in a dark room. A fraction collector with metal-free, pre-weighed polypropylene tubes was used to collect the dialysates over the course of 7 days. The sample weight for each tube was recorded. The solubilized amount of Ti in the fractions were all below the instrument limit of detection (~10 ng/mL) by atomic emission spectroscopy (Beckman Spectraspan V, Fullerton, CA).

### Quantification of TiO_2_ NPs in lung tissues

TiO_2_ NP-exposed rats were euthanized with an overdose of 2, 2, 2-tribromoethanol (Avertin; 25 mg/100 g body weight, i.p.); the pelts were removed to eliminate possible transfer of TiO_2_ to the lung tissue from the animals’ fur as previously described [77]. Lung tissues were harvested immediately following exposure (ILB), 24 hr and 7 days post exposure by excising the lung above the bifurcation of the main bronchi. Tissue samples were dried at 85°C and then ashed at low temperature (50–100°C) in a solid state plasma asher (March Instruments Inc., Concord, CA), in which organic material is gently oxidized to CO_2_, leaving only TiO_2_ and inorganic ash. Samples were then fused with sodium carbonate/sodium borate (2:1; Sigma, St. Louis, MO) at 1500°C in platinum crucibles for 20 min or until a clear melt was formed. The melt was cooled and then dissolved in 2.5 N sulfuric acid and diluted 1:2 with ultra pure water. The concentration of Ti was quantified using atomic emission spectroscopy and the mass of TiO_2_ in each sample was then determined stoichiometrically. Control and naïve animals were found to have background levels of TiO_2_ in the lung below the instrument limit of detection for atomic emission spectroscopy (~10 ng/mL).

### Cellular and biochemical parameters in bronchoalveolar lavage fluid

Separate groups of rats were euthanized at 4, 8, 24 hr and 7 days after instillation or after the beginning of the inhalation exposures with an overdose of Avertin followed by exsanguination. The lung/heart block was excised and excess tissue removed prior to the lungs being lavaged with sterile, 0.9% saline (5 × 5 mL), keeping the first two lavage supernatants separate from the remaining ones following centrifugation (10 min, 350 × *g*, 4°C). BAL cell viability (trypan blue exclusion), number, and the percentage of different cell types (Hema 3®; Fisher Scientific, Kalamazoo, MI) were determined. Total protein concentration was measured as an indicator of cytotoxicity and epithelial barrier permeability with the bicinchoninic acid (BCA) assay using reagents purchased from Thermo Scientific (Rockford, IL). Lactate dehydrogenase and β-glucuronidase activities, as indicators of cell membrane and lysosomal membrane integrity, respectively, were determined using reagents from Sigma.

### Preparation of lung homogenates

Flash frozen, right lung tissues were homogenized on ice for 30 sec in 4.5 mL of radioimmunoprecipitation assay (RIPA) buffer, comprised of reagents from Sigma (50 mM Tris–HCl, 150 mM NaCl , 0.25% deoxycholic acid, 1 mM EDTA, 0.1 mM PMSF) and Roche (Indianapolis, IN; 1% nonindet P-40, 10 μg/mL aprotinin and 10 μg/mL leupeptin). Samples were centrifuged for 1 hr at 19,800 × *g* and 4°C in 50 mL, round PPCO tubes (Nalgene, Rochester, NY). The protein content of the supernatants was measured using the BCA assay.

### Measurements of inflammatory mediators

The lung homogenate and the BAL fluid supernatants were used for measuring MCP-1, MIP-2 (CXCL2/CINC-3), TNF-α, and IL-10 levels by ELISA using antibodies and protocols from BD Biosciences (San Diego, CA), R&D Systems (Minneapolis, MN), eBioscience (San Diego, CA) and Invitrogen (Frederick, MD), respectively. HO-1 in BALF cell pellet lysates (lysis buffer: 1.25x protease inhibitor cocktail P2714 from Sigma, 1% Triton X and 0.2 mM PMSF) and supernatants were also assessed by ELISA (Enzo Life Sciences, Farmingdale, NY).

### Data analysis

The dosimetry results were analyzed for time related changes from ILB by one-way ANOVAs for both exposure methods. Differences between the exposure methods at the same post-exposure time points were assessed by a Student’s t-test. Response endpoints for the high dose single exposures were analyzed by two-way ANOVA to detect differences over time and between exposure methods. Separate control groups for every post-exposure time point were not included due to ethical reasons, specifically to limit the total number of animals used in the study, as it was not considered likely that within-exposure-method responses in controls would change consistently over the time course of this study. Therefore, controls were evaluated only at 24 hr post exposure (the endpoint of highest possible acute inflammation) and 4 hr post (the earliest time point assessed for the onset of the inflammatory responses). Responses in controls were only found to have significant time related differences within inhalation exposure for cell viability, BALF MCP-1 and homogenate MIP-2 and within instillation exposure for BALF protein, homogenate MIP-2, IL-10 and BALF pellet HO-1. Because there was no consistency in terms of which time point had higher values and the small differences were not likely to be biologically significant, the controls were pooled. The low dose response and repeated exposure data were analyzed by two-way ANOVAs with exposure method and dose of TiO_2_ as the main factors. Data were appropriately transformed if analyses of residuals suggested deviations from the assumptions of normality and equal variance. Two outliers were identified based on analyses of residuals (β-glucuronidase and BALF protein) and were removed from figures, tables and corresponding ANOVA tests. All comparisons were considered statistically significant when *p* < 0.05.

## Competing interests

The authors declare that they have no competing interests.

## Authors’ contributions

BB conceived of the study, participated in its design, characterized the material in liquid suspension, performed exposures, sample collections, acquisition of data, statistical analyses and drafted the manuscript. NC, PW-M and AK performed the exposures, sample collection, and acquisition of data. RG conceived the design for the dosimetry experiments and performed the analytical measurements of Ti content, assisted with exposures and characterization of the TiO_2_ aerosol. GO and AE provided advice for the study, participated in its design, and assisted with data interpretation and revision of the manuscript. All authors read and approved the final manuscript.

## Supplementary Material

Additional file 1: Figure S1 Suspension and Sonication Time Effects on the Inflammatory Response. The neutrophil response 24 hr post intratracheal instillation with TiO_2_ that was: **(A)** suspended in DM *(red)* or saline *(green)* and cup horn sonicated for 5 sec; **(B)** suspended in saline and cup horn sonicated for 5 sec *(solid green)*, 5 min *(green dots)*, or 30 min *(green checkers)*. Bars are group means (n = 5) ± SE and are shown as a percentage of corresponding controls (DM or saline). In *A* the *indicate a significant increase from corresponding control and Φ indicates a significant difference between dispersants as determined by a two-way ANOVA (p < 0.05). In *B* the *indicate significant increases from corresponding control and Ω indicate significant differences from the other two sonication times as determined by a two-way ANOVA (p < 0.05). Click here for file
