# Supplementary figures and images for: Equivalent titanium dioxide nanoparticle deposition by intratracheal instillation and whole body inhalation: the effect of dose rate on acute respiratory tract inflammation
Source: Part Fibre Toxicol. 2014 Jan 24;11:5. doi: 10.1186/1743-8977-11-5 (PMC3905288; doi:10.1186/1743-8977-11-5)

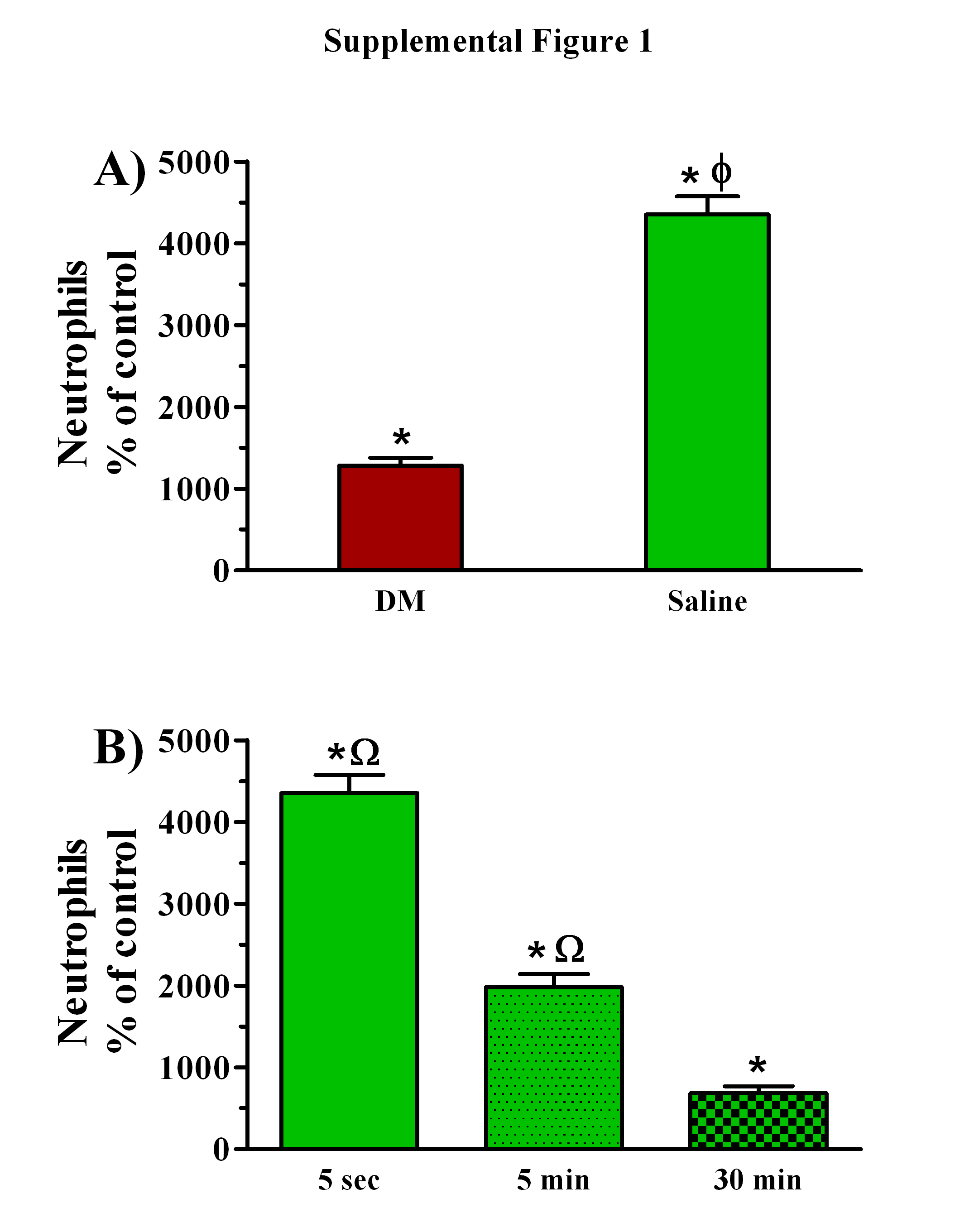

Supplement: Additional file 1: Figure S1 — Suspension and Sonication Time Effects on the Inflammatory Response. The neutrophil response 24 hr post intratracheal instillation with TiO2 that was: (A) suspended in DM (red) or saline (green) and cup horn sonicated for 5 sec; (B) suspended in saline and cup horn sonicated for 5 sec (solid green), 5 min (green dots), or 30 min (green checkers). Bars are group means (n = 5) ± SE and are shown as a percentage of corresponding controls (DM or saline). In A the *indicate a significant increase from corresponding control and Φ indicates a significant difference between dispersants as determined by a two-way ANOVA (p < 0.05). In B the *indicate significant increases from corresponding control and Ω indicate significant differences from the other two sonication times as determined by a two-way ANOVA (p < 0.05). [file 1743-8977-11-5-S1.doc]
